# Clonal expansion of global pneumococcal sequence cluster 3 within serotype 8 after 13-valent pneumococcal conjugate vaccine introduction, South Africa

**DOI:** 10.1099/mgen.0.001737

**Published:** 2026-06-02

**Authors:** Cebile Lekhuleni, Kedibone Ndlangisa, Ana D. Sanches Ferreira, Hsueh-­Chien R. Cheng, Oliver Lorenz, Jackie Kleynhans, Linda de Gouveia, Happy Skosana, Vanessa Quan, Amelieke J.H. Cremers, Paulina A. Hawkins, Sopio Chochua, Lesley McGee, Stephen D. Bentley, Susan Meiring, Cheryl Cohen, Stephanie W. Lo, Anne von Gottberg, Mignon du Plessis

**Affiliations:** 1 Centre for Respiratory Diseases and Meningitis, National Institute for Communicable Diseases, a division of the National Health Laboratory Service, Johannesburg, South Africa; 2 School of Pathology, Faculty of Health Sciences, University of the Witwatersrand, Johannesburg, South Africa; 3 Institute of Microbiology and Infection, College of Medicine and Health, University of Birmingham, Edgbaston, Birmingham, UK; 4 Parasites and Microbes Programme, The Wellcome Sanger Institute, Wellcome Genome Campus, Hinxton, Cambridge, UK; 5 School of Biological Sciences, Queen’s University Belfast, Belfast, UK; 6 School of Public Health, Faculty of Health Sciences, University of the Witwatersrand, Johannesburg, South Africa; 7 Division of Public Health Surveillance and Response, National Institute for Communicable Diseases, a division of the National Health Laboratory Service, Johannesburg, South Africa; 8 Department of Laboratory Medicine, Laboratory of Medical Immunology, Radboudumc Community for Infectious Diseases, Nijmegen, The Netherlands; 9 Department of Fundamental Microbiology, Faculty of Biology and Medicine, University of Lausanne, Lausanne, Switzerland; 10 Division of Bacterial Diseases, National Center for Immunization and Respiratory Diseases, Centers for Disease Control and Prevention, Atlanta, GA 30329, USA; 11 European Molecular Biology Laboratory-European Bioinformatics Institute, Cambridge, UK; 12 The Great Ormond Street Institute of Child Health, University College London, London, UK; 13 Division of Medical Microbiology, Department of Pathology, Faculty of Health Sciences, University of Cape Town, Cape Town, South Africa

**Keywords:** global pneumococcal sequence cluster (GPSC), pneumococcal conjugate vaccine (PCV), serotype 8, *Streptococcus pneumoniae*

## Abstract

**Background**. Serotype 8 has emerged as a common non-vaccine serotype causing invasive pneumococcal disease (IPD) in children and adults globally after the introduction of pneumococcal conjugate vaccines (PCV). We aimed to determine serotype 8 incidence and genomic epidemiology in South Africa.

**Methods**. From national, laboratory-based surveillance for IPD in South Africa, we calculated the incidence of serotype 8 and global pneumococcal sequence cluster 3 (GPSC3) before and after PCV introduction. We performed phylodynamic analysis of genomes from clonal complex (CC) 53 and described South African isolates in the context of a global collection of serotype 8 and GPSC3 genomes.

**Results.** Compared to the pre-PCV period, serotype 8 incidence increased in the late-PCV13 period among children <5 years [incidence rate ratio (IRR) 2.3, 95% confidence interval (CI) 1.3–4.1] and adults >64 years (2.8, 1.1–8.3). Similarly, GPSC3 rates increased among children <5 years (IRR 2.4, 95% CI 1.4–4.3) and individuals of all ages (1.7, 1.3–2.1). Serotype 8 CC53 population dynamics predicted at least five clonal expansions since the most recent common ancestor in 1929 (credible interval 1919–1937), with future predictions showing increases in the effective population size despite remaining largely susceptible to first-line treatment. Serotype 8 CC53 isolates from South Africa had a clonal structure when contextualized within a global collection of serotype 8 and GPSC3 genomes.

**Conclusion.** Serotype 8 invasive disease incidence increased in South Africa after PCV introduction. Predictions showed sustained increases over time. Since South Africa switched to a lower-valency PCV10 (Pneumosil, Serum Institute of India) that does not include serotype 8 in 2024, continued surveillance will be crucial to inform PCV policy-making.

**Impact Statement**

Non-13-valent pneumococcal conjugate vaccine serotype 8 has emerged as an important cause of invasive pneumococcal disease in children and adults following the introduction of PCV in South Africa. Previous studies have shown that PCV use led to an increase in the incidence of invasive disease caused by pre-existing non-vaccine type pneumococcal clones and lineages. Our study corroborates these findings using surveillance data spanning 16 years and describes the genomic epidemiology of serotype 8 lineages in South Africa, placing these in a global context. Our findings contribute to the understanding of pneumococcal genomic population structure in response to vaccine use.

## Data Summary

Genome sequences were deposited in the European Nucleotide Archive, and accession numbers and metadata are included in the supplementary data file (Data S1). Additional genomes from this study are available on the Monocle Database available at https://data.monocle.sanger.ac.uk/. Sample and public names are included in Data S1.

## Background

Pneumococcal conjugate vaccines (PCV) have effectively reduced vaccine serotype invasive pneumococcal disease (IPD) across countries of different income economies [1,3]. However, the incidence of non-vaccine serotype disease increased in several countries following routine PCV use [4,6]. In Australia, Finland, France, the Netherlands and Norway, non-13-valent PCV (non-PCV13) serotypes, especially serotype 8, contributed substantially to IPD in adults following vaccine introduction [7,8]. In South Africa, the seven-valent pneumococcal conjugate vaccine (PCV7) was introduced into the childhood immunization programme in 2009 and was replaced by PCV13 in 2011 [1,9]. Consequently, non-vaccine serotypes became the most common cause of invasive disease and accounted for 73% of IPD, of which serotype 8 contributed 23% among individuals <18 years during the 2015–2020 period [10]. Among individuals aged ≥65 years, serotype 8 disease increased notably in 2019 from a proportion of 8% to 14% when compared to the baseline period (2005–2008), with this increase partially offsetting the overall benefits of IPD reduction due to PCV use [11].

Before PCV introduction, sequence type (ST) 53 was the predominant serotype 8 genotype in South Africa [12]. In Madrid, Spain, the ST53 clone of serotype 8 drove the increase in non-vaccine serotype IPD after PCV13 introduction [13]. Similarly, in Denmark and in the Netherlands, serotype 8 ST53 was the dominant clone both before and after the introduction of PCVs and contributed to the increase in serotype 8 incidence in the elderly after PCV13 introduction [8,14]. Population snapshot studies conducted as part of the global pneumococcal sequencing (GPS) project have shown that serotype 8 clonal complex (CC) 53 falls within the globally disseminated global pneumococcal sequence cluster 3 (GPSC3) lineage [15]. In South Africa, GPSC3 was the most common cause of non-vaccine serotype invasive disease in children <3 years in the 3 years following PCV13 introduction (2011–2014) [15] and increased significantly (0.73 to 1.32 cases per 100,000 population, *P*<0.008) in children <5 years from the pre-PCV (2005–2008) to the late-PCV13 (2015–2020) periods [10]. Although the global contextualization of predominant lineages such as GPSC3 is essential for understanding lineage-specific invasive disease contributions, country-specific analyses are equally important for understanding localized expansion mechanisms and disease burden. Our study aimed to evaluate the expansion of serotype 8 in South Africa and to describe and place circulating genotypes and GPSC lineages into a global context.

## Methods

### Invasive pneumococcal disease surveillance in South Africa

We included invasive *Streptococcus pneumoniae* collected as part of GERMS-SA, a national, laboratory-based surveillance programme, a precursor of which was initiated in 1999 across all nine South African provinces [16]. Over 200 participating laboratories submit isolates and patient data to the reference laboratory at the National Institute for Communicable Diseases (NICD). Approximately 30 sentinel sites submit additional demographic and clinical data, such as HIV serostatus, clinical syndrome and outcome [17]. IPD was defined as the detection of *S. pneumoniae* from a normally sterile-site specimen [e.g. blood, cerebrospinal fluid (CSF), joint fluid and pleural fluid], either positive by culture, PCR or latex agglutination.

### Routine phenotypic characterization

The Quellung reaction using serotype-specific antiserum (Statens Serum Institut, Copenhagen, Denmark) [18] was used for serotyping viable pneumococcal cultures. PCR was used for serotyping non-viable cultures and sterile-site specimens [19,21]. Antimicrobial susceptibility testing (AST) was performed as previously described [22]. Briefly, disc diffusion was used to screen for antimicrobial susceptibility for all isolates. Minimum inhibitory concentrations (MICs) were determined by agar dilution or Etest from 2005 to 2008. From 2009 onwards, broth microdilution was routinely used, and MICs were interpreted according to the 2018 Clinical and Laboratory Standards Institute guidelines [23].

### Whole-genome sequencing and molecular characterization

The sampling strategy for sequencing was previously described [10]. Approximately 300 IPD isolates of all serotypes were randomly selected from GERMS-SA per year (2005–2020) from individuals of all ages, stratified by age category (0–2, 3–5 and >5 years). Due to high disease burden, sampling was biassed towards the ≤2 year olds, an age group targeted for vaccination. Sequencing was performed using Illumina (HiSeq, NextSeq or NovaSeq, Illumina, San Diego, CA, USA). Genomic data were processed as previously described [24]. SeroBA version 1.0.1 was used to determine *in silico* serotype [25]. From a total of 4,834 sequenced isolates from South Africa, 391 (8.1%) were serotype 8 and were included in this study. For these, Quellung results and PCR serotyping data were 100% concordant with *in silico* serotyping. Raw data accession numbers for the European Nucleotide Archive are listed in Data S1 (available in the online Supplementary Material).

MLSTcheck (version 2.1.1706216) [26] was used to determine seven-locus STs. Isolates sharing at least six of the seven multi-locus sequence type (MLST) housekeeping genes were grouped into CCs and were defined as single-locus variants. GoeBURST in PHYLOViZ (version 2.0) was used to create an MLST minimum-spanning tree showing allelic profile relationships [27]. Using whole genomes, GPSCs were assigned using PopPUNK (version 2.4.0) and the GPS reference database version 6 (https://www.pneumogen.net/gps/#/gpsc) [28]. Antimicrobial resistance determinants for penicillin (*pbp*1a, *pbp*2b and *pbp*2x), erythromycin (*ermB* and *mefA*), tetracycline (*tet*M and *tet*S/M), cotrimoxazole (*folA*_I100L, *folP*_aa_insert_57–70) and *in silico* MICs were determined as previously described [29]. The correlation between phenotypic and *in silico* MIC has also been previously described for South African data [10]. Genome annotation was done using Prokka (version 1.14.5-c2) [30]. Serotype 8 capsular locus sequences (CPS) were extracted using blast (version 2.15.0) and examined for genetic variations by CPS alignment. The serotype 8 CPS reference CR931644 was used to evaluate gene content differences and to check for disruptive mutations. CPS sequences for serotypes expressed by GPSC3 were also extracted using blast and Clinker [31] was used for *cps* loci visualization.

### Phylogenetic analysis

A total of 686 serotype 8 genomes were analysed: 391 from South Africa (2005–2020) and 295 publicly available genomes from 27 other countries in the GPS collection (1991–2020) by mapping against the *S. pneumoniae* reference genome ATCC 700669 (National Center for Biotechnology Information accession code FM211187) using Smalt version 0.7.4 (https://github.com/rcallahan/smalt). The pseudo-genome alignment was reduced to an alignment of variant sites using the SNP-sites tool (version 2.5.1--hed695b0_0) [32]. The single nucleotide polymorphism (SNP) alignment was used to construct a phylogenetic tree using the generalized time-reversible (GTR) model in FastTree2 (version 2.1.10 h470a237_2) [33]. A lineage-specific phylogeny of 749 GPSC3 isolates (including non-serotype 8 isolates of the same lineage) from South Africa (*n*=447, 2005–2020) and 14 other countries in the GPS collection (*n*=302, 1997–2020) was constructed by mapping Illumina reads against a GPSC3 reference (*S. pneumoniae* AP200 accession code CP002121) using Burrows–Wheeler Alignment version 0.7.17-r1188 [34]. Gubbins (version 3.2.1) was used to identify recombination events among serotypes expressed by GPSC3 and to construct a recombination-free phylogeny using the GTR model in RAxML [35,36]. Microreact was used to visualize phylogenies [37].

### Clonal expansion analysis of CC53

A recombination-free phylogeny of a subset (*n*=219) of serotype 8 CC53 genomes from South Africa that resulted in a significant temporal signal was used for Bayesian inference of ancestral dates using BactDating to perform root-to-tip regression [38]. The CaveDive R package that uses a coalescent-based model to reconstruct effective population size over time was then used to analyse the expansion dynamics of the CC53 clone using reversible-jump Markov chain Monte Carlo (MCMC) inference ran for 100 million MCMC iterations using default settings [39]. Carrying capacity was defined as the maximum population size of an expanding subpopulation [39].

### Statistical analysis

Statistical analysis was performed using all reported serotype 8 cases from South Africa, 2005–2020. We defined PCV periods as: pre-PCV (2005–2008), PCV7 (2009–2010), early-PCV13 (2011–2014) and late-PCV13 (2015–2020). Mid-year population from Stats SA (https://www.statssa.gov.za/) was used as denominator to calculate serotype 8 IPD incidence (per 100,000 population) for age groups: 0–4, 5–14, 15–24, 25–44, 45–64 and >64 years by comparing the average number of cases in the pre-PCV period with those in the late-PCV13 period. GPSC3 incidence was calculated using imputed counts by assuming that the proportion of GPSC3 from the serotype 8 genomes from South Africa by year and by age group was similar to that of the total number of serotype 8 isolates from IPD surveillance. We used the Wilcoxon rank sum test to compare the GPSC3 mutation rate with pre-existing non-GPSC3 mutation rates previously determined by Gladstone and colleagues [24]. Statistical analyses were performed in Stata 18.0 (StataCorp, College Station, TX, USA) and R version 4.2.1.

## Results

### NationalIPD surveillance, South Africa

A total of 54,199 IPD cases were reported from 2005 through 2020, of which 39,377 (72.7%) had serotyping data (Fig. S1). Serotype 8 accounted for 3.3% (453/13,725), 3.0% (202/6,657), 5.6% (521/9,308) and 13.4% (1,297/9,687) of IPD cases during the pre-PCV, PCV7, early-PCV13 and late-PCV13 periods, respectively. Of the 2,473 serotype 8 cases identified during the study period, age was recorded for 2,429 (98.2%), of which 2,170 (89.3%) had viable isolates. Of these, 391 (18.0%) isolates from individuals of all ages were sequenced (Table 1).

**Table 1.** Serotype 8 invasive pneumococcal disease isolates stratified by vaccine period (pre-PCV, PCV7, early-PCV13 and late-PCV13) among individuals of all ages in South Africa, 2005–2020, *N*=2,429 (number of cases with known age), *n*=391 (number of sequenced isolates)

	Pre-PCV (2005–2008)		PCV7 (2009–2010)		Early-PCV13 (2011–2014)		Late-PCV13 (2015–2020)		Total	
	*N*	n seq. (%)	*N*	n seq. (%)	*N*	n seq. (%)	*N*	n seq. (%)	*N*	n seq. (%)
	439	30 (7)	197	9 (5)	504	83 (17)	1289	269 (21)	2429	391 (16)
**Year**										
2005	100	8 (8)								
2006	119	8 (7)								
2007	114	5 (4)								
2008	106	9 (9)								
2009			97	5 (5)						
2010			100	4 (4)						
2011					104	10 (10)				
2012					124	18 (15)				
2013					137	28 (20)				
2014					139	27 (19)				
2015							203	39 (19)		
2016							226	47 (21)		
2017							257	40 (16)		
2018							228	44 (19)		
2019							227	44 (19)		
2020							148	55 (37)		
**Province**										
Eastern Cape	20	2 (10)	10	0 (0)	38	9 (24)	120	24 (20)	188	35 (19)
Free State	23	0 (0)	16	0 (0)	30	7 (23)	62	17 (27)	131	24 (18)
Gauteng	243	17 (7)	100	7 (7)	244	43 (18)	472	107 (23)	1059	174 (16)
KwaZulu-Natal	35	0 (0)	15	0 (0)	47	10 (21)	67	13 (19)	164	23 (14)
Limpopo	6	0 (0)	6	0 (0)	6	1 (17)	58	13 (22)	76	14 (18)
Mpumalanga	12	1 (8)	5	0 (0)	18	4 (22)	51	9 (19)	86	14 (16)
Northern Cape	5	0 (0)	6	1 (17)	11	0 (0)	35	6 (17)	57	7 (12)
North West	21	1 (5)	3	0 (0)	27	2 (7)	41	10 (24)	92	13 (14)
Western Cape	56	9 (16)	35	1 (3)	82	7 (9)	374	70 (19)	547	87 (16)
Unknown	18	0 (0)	1	0 (0)	1	0 (0)	9	0 (0)	29	0 (0)
**Sex**										
Male	236	15 (6)	104	4 (4)	243	43 (18)	661	146 (22)	1244	208 (17)
Female	196	15 (8)	90	5 (6)	255	37 (15)	619	120 (19)	1660	177 (11)
Unknown	7	0 (0)	3	0 (0)	6	3 (50)	9	3 (33)	25	6 (24)
**Age**										
0–4 years	75	14 (19)	41	4 (10)	114	54 (47)	293	146 (50)	523	218 (42)
5–14 years	22	1 (5)	12	1 (8)	20	4 (20)	39	4 (10)	93	10 (11)
15–24 years	22	0 (0)	19	0 (0)	23	1 (4)	58	10 (17)	122	11 (9)
25–44 years	216	14 (7)	74	2 (3)	208	19 (9)	454	56 (12)	952	91 (10)
45–64 years	81	1 (1)	44	2 (5)	107	3 (3)	315	37 (12)	547	43 (8)
>64 years	23	0 (0)	7	0 (0)	32	2 (6)	130	16 (12)	192	18 (9)
**Specimen type**										
Cerebrospinal fluid	170	11 (7)	72	2 (3)	231	46 (20)	448	110 (26)	921	169 (18)
Blood	253	19 (8)	109	7 (6)	242	36 (15)	786	154 (20)	1390	216 (16)
Others	16	0 (0)	16	0 (0)	31	1 (3)	55	5 (9)	118	6 (5)

**Fig. 1.**
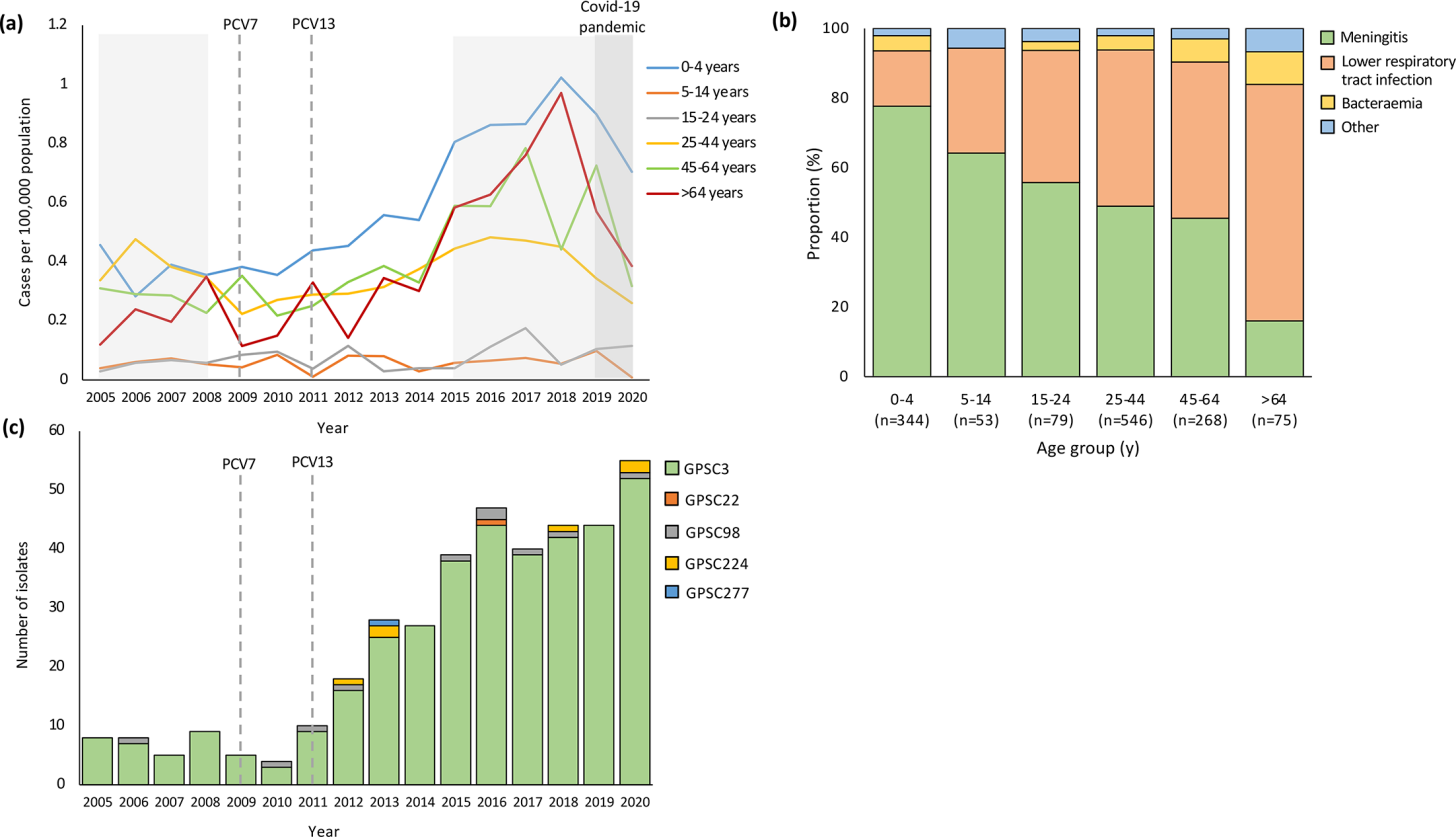
Serotype 8 trends and characteristics, 2005–2020, South Africa. (**a**) Incidence of serotype 8 invasive pneumococcal disease reported by year and by age group (*N*=2,429). PCV7 and PCV13 introduction years are shown by dotted lines. Pre-PCV (2005–2008) and late-PCV13 (2015–2020) periods are highlighted in light grey; the COVID-19 pandemic is highlighted in dark grey. (**b**) Known serotype 8 clinical syndromes by age group (*n*=1,365 of 2,429). (**c**) Number of sequenced serotype 8 isolates (*n*=391) by year, stratified by global pneumococcal sequencing clusters (GPSC). PCV7 and PCV13 introduction years are shown by dotted lines.

Serotype 8 IPD incidence increased significantly in the late-PCV13 period among children <5 years [incidence rate ratio (IRR) 2.3, 95% confidence interval (CI) 1.3–4.1], adults 45–64 years (2.1, 1.2–3.7) and adults >64 years (2.8, 1.1–8.3), compared to the pre-PCV period (Fig. 1a and Table S1). The majority of serotype 8 cases among children <5 years were detected in CSF (310/523, 59.3%), corresponding to the higher proportion (267/344, 77.6%) of children with meningitis clinical syndrome (Table 1, Fig. 1b). In contrast, among adults ≥25 years, the majority of serotype 8 cases were detected from blood which accounted for 84.4% (162/192) of cases in adults >64 years, correlating to the higher proportion (58/75, 77.3%) of lower respiratory tract infection and bacteraemia syndromes in this age group (Table 1, Fig. 1b).

Serotype 8 isolates were largely susceptible to penicillin (98.1%, MIC_90_=0.03 μg ml^−1^), erythromycin (99.2%, MIC_90_=0.06 μg ml^−1^), tetracycline (98.0%, MIC_90_=2 μg ml^−1^) and cotrimoxazole (95.7%, MIC_90_=0.5 μg ml^−1^). When comparing phenotypic AST results among the 391 sequenced isolates from South Africa with *in silico* AMR predictions, concordances of 98.2% (*n*=384), 99.5% (*n*=389), 99.0% (*n*=387) and 96.2% (*n*=376) were observed for penicillin, erythromycin, tetracycline and cotrimoxazole, respectively. Due to low discordance that is within previously described ranges [40,41], we did not exclude discordant isolates.

### Serotype 8 genotypes and GPSC lineages, South Africa

Using seven-locus MLST, 384/391 (98.2%) serotype 8 isolates were grouped into five CCs (CC53, CC1012, CC1480, CC3406 and CC10588). The remaining seven isolates differed at five or more MLST loci. GPSC3, composed of CC53 (*n*=371, 94.9%) and CC1012 (*n*=2, 0.5%), was the predominant serotype 8 lineage. The remaining 18 isolates were grouped into four GPSCs and accounted for 4.6% of the isolates (Figs 1c and S2). We observed significant increases in the incidence of GPSC3 IPD among children <5 years (IRR 2.4, 95% CI 1.4–4.3) and individuals of all ages (IRR 1.7, 95% CI 1.3–2.1).

### Clonal expansion of CC53 (GPSC3), South Africa

**Fig. 2.**
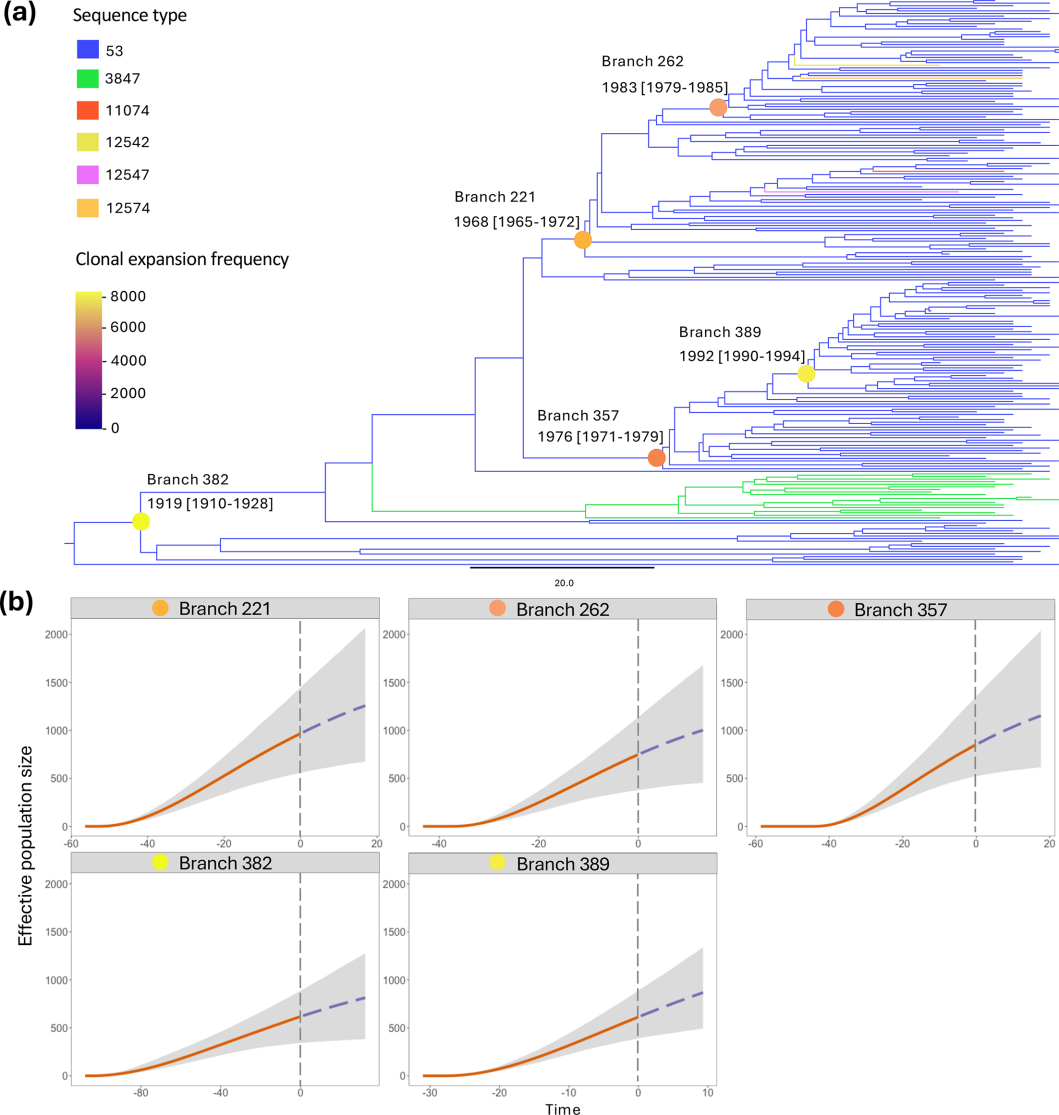
CC53 expansion dynamics among genomes from South Africa (*N*=219) inferred using CaveDive. (**a**) Time-calibrated phylogeny showing clonal expansion branches within CC53. Clonal expansion nodes are represented by coloured circles with corresponding per-branch frequencies shown by the colour scale on the left. Phylogeny clades are coloured by sequence type. The phylogenetic tree is shown in increasing node order. (**b**) Expansion dynamics of CC53 subpopulations (expansion branches) showing the effective population sizes over time. Time zero (grey dashed vertical line) represents the year 2020 (end of the study period). The orange solid line represents median past population dynamics, and the purple dashed line represents the predicted population dynamics. The grey shaded area represents 95% credible intervals.

The CC53 root-to-tip regression analysis showed a significant temporal signal (*R^2^*=0.20 and *P*<0.001) permitting coalescent analysis. The most recent common ancestor was around 1929 [credible interval (CI) 1919–1937] (Fig. S3a). The 95% CIs for inferred ancestral dates are shown in Fig. S3b. CaveDive analysis estimated at least five clonal expansions within the CC53 clone (Fig. 2a), all of which occurred before the introduction of PCV with the most recent expansion occurring around 1992 (CI 1990–1994). Predicted future population dynamics of clonal expansion branches showed steady increases in the effective population sizes of CC53 subpopulations over the next 10 years, at least (starting from 2020), in the absence of a serotype 8-targeting intervention (Fig. 2b).

### Global distribution of serotype 8 genotypes and GPSC lineages

**Fig. 3.**
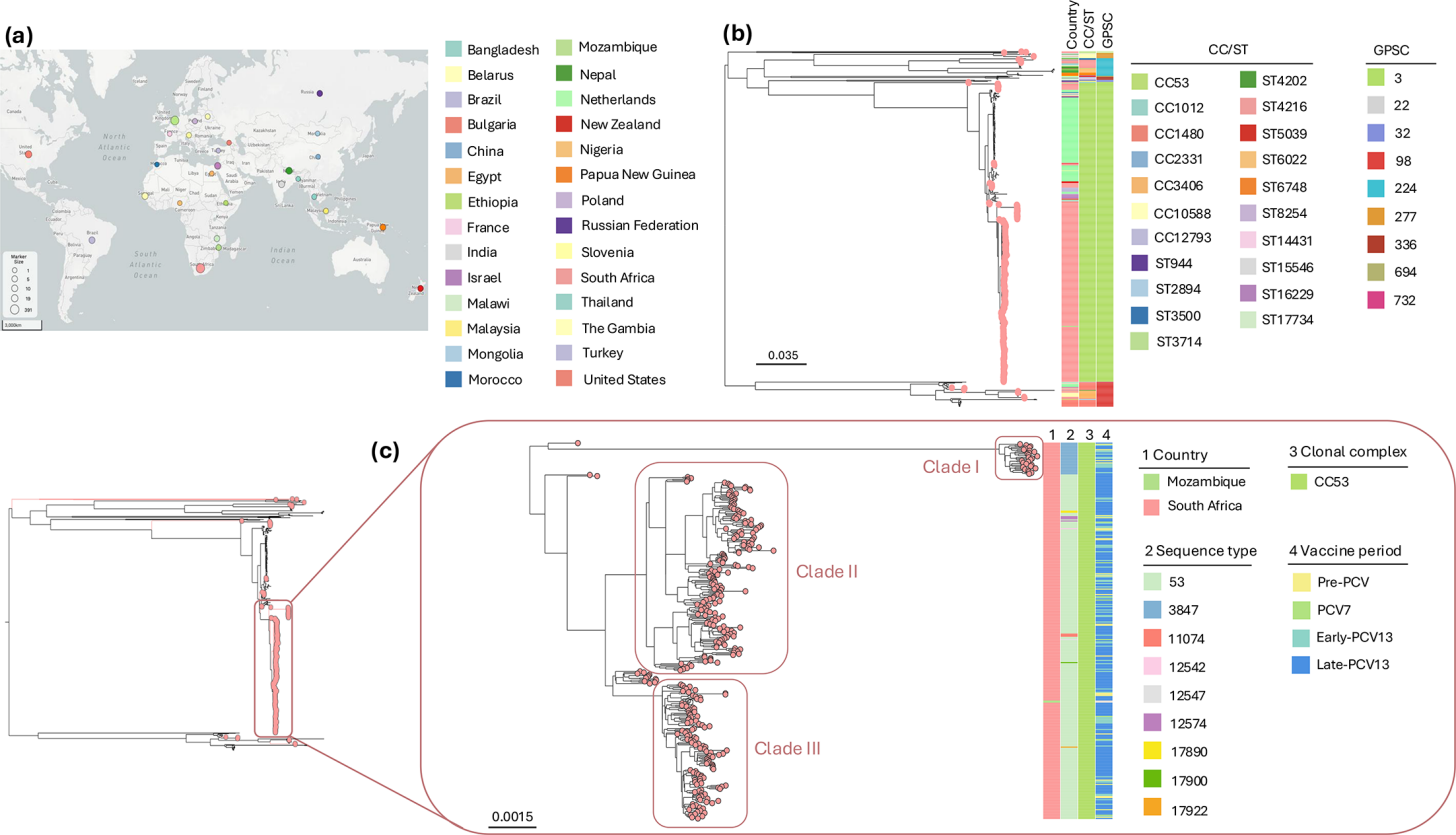
Contextualization of South African serotype 8 genomes. (**a**) Distribution of serotype 8 genomes from South Africa (*n*=391) and 27 other countries (*n*=295). The coloured circles on the map are proportional to the number of sequenced isolates by country. (**b**) Phylogeny of all serotype 8 genomes (*N*=686) with only South African leaf nodes highlighted. (**c**) Expanded South African serotype 8 subtree (*n*=343) showing country-specific genomic structure. Distinct clades (I to III) are circled and labelled in pink. *Microreact instance link for phylogeny visualization: Serotype_8_global_phylogeny.

Among 686 serotype 8 isolates (391 from South Africa and 295 from 27 countries, Table S2), 640 isolates (93.3%) were grouped into 7 CCs with varying predominance by continent. The remaining 46 isolates had distinct STs that did not belong to any CC. CC53 was predominant among isolates from Africa [374/413, (90.6%)], Europe [188/203 (92.6%)] and Latin America [9/10 (90.0%)]; the majority of isolates from North America were CC1480 [14/19 (73.7%)]. Serotype 8 isolates from Asia were mainly ST4216 (*n*=8), ST6022 (*n*=8), CC10588 (*n*=4) and CC12793 (*n*=5), together contributing to 83.3% (25/30) of the isolates. ST6748 and CC53 together contributed to 90.9% (10/11) of genotypes from Oceania in this collection. Despite the global dissemination of serotype 8 (CC53), we observed a geographical structure within the CC53 clade (Fig. 3a and b). CC53 isolates from South Africa clustered together in a clade, within which three subclades (clades I to III) were identified (Fig. 3b and c). Two ST53 isolates from Mozambique clustered within clade III (Fig. 3c). Overall, the serotype 8 *cps* locus was largely conserved among the 686 serotype 8 isolates from the global collection, although isolates with >10 non-disruptive SNPs per gene were observed for three (*wzg*, *wchA* and *wzx*) of the 12 *cps* genes at proportions of 9.3% (64/686), 88.8% (608/686) and 88.6% (608/686), respectively, and were distributed across different specimen types, syndromes, CCs and GPSCs (Fig. S4).

**Fig. 4.**
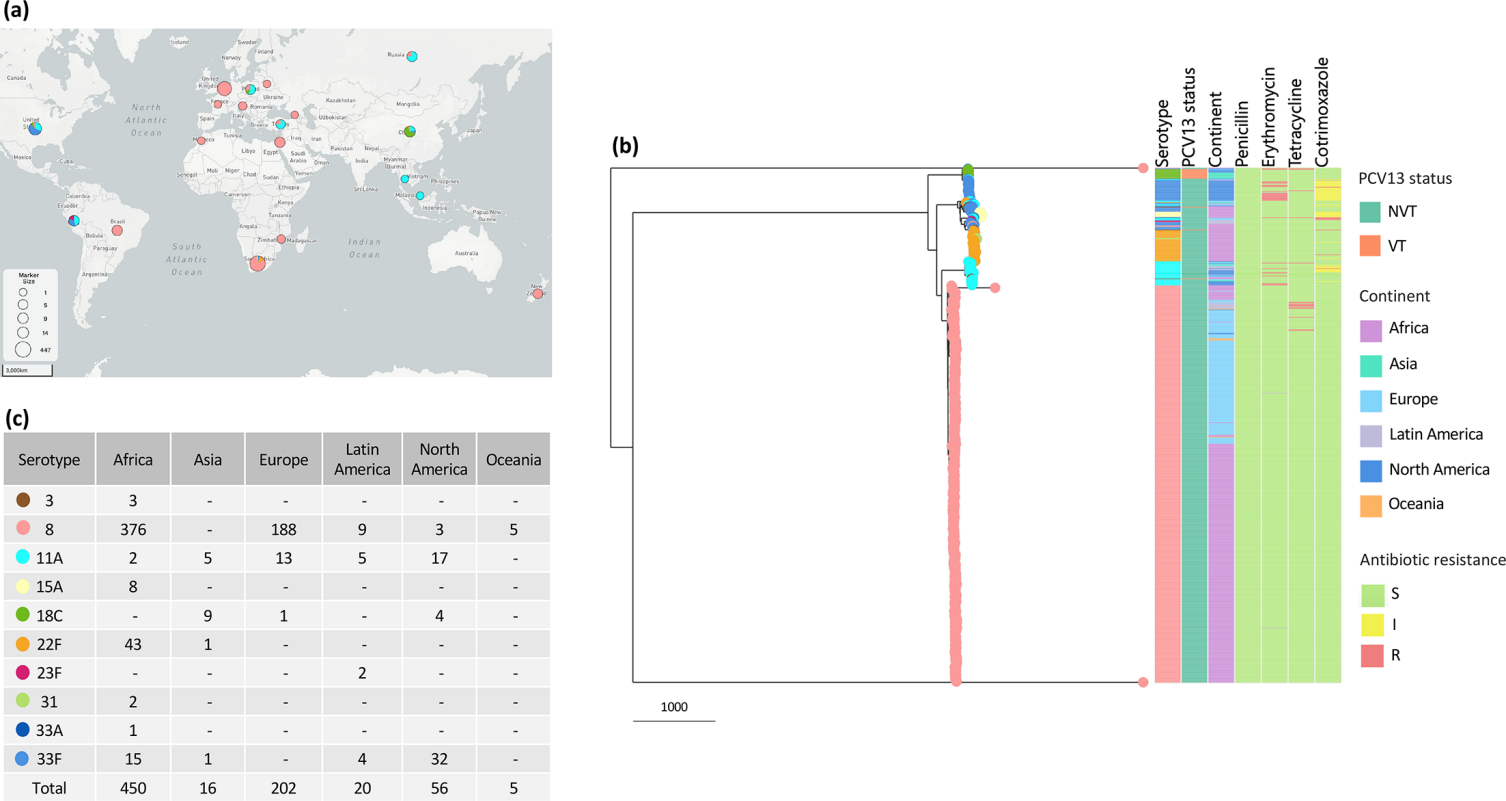
Global distribution of GPSC3 lineage (*N*=749). (**a**) The pie charts are coloured by serotypes expressed by GPSC3 and are proportional to the number of isolates in each country. (**b**) The phylogeny shows the genetic relatedness of GPSC3 serotypes globally, with metadata inclusive of *in silico* antimicrobial resistance profiles for the antibiotics of interest. (**c**) Shows the distribution of serotypes by continent.

Globally, we identified nine GPSC lineages that expressed serotype 8 in this collection. GPSC3 (CC53 and CC1012) was the most predominant lineage, accounting for 84.7% (581/686) of the isolates and circulated on all continents, except Asia (Fig. 4 and Table S3). GPSC98 (CC1480, CC3406, ST2894 and ST4202) was the second most common lineage (6.7%, *n*=47), and similar to GPSC3, we observed global dissemination, except in Asia. Serotype 8 isolates from Asia were predominantly expressed by GPSC224 (17/30, 56.7%), of which ST4216 and ST6022 equally comprised 16/17 (94.1%) of the isolates (Fig. 3b, Tables S3 and S4). Each of GPSC22, GPSC32, GPSC277, GPSC336, GPSC694 and GPSC732 expressed at most ten isolates in this collection and accounted for 3.5% (24/686) of the isolates (Table S3). GPSC3 (serotype 8) remained largely susceptible to penicillin, erythromycin, tetracycline and cotrimoxazole (Fig. 4b and c).

### Analysis of the predominant serotype 8-expressing lineage – GPSC3

**Fig. 5.**
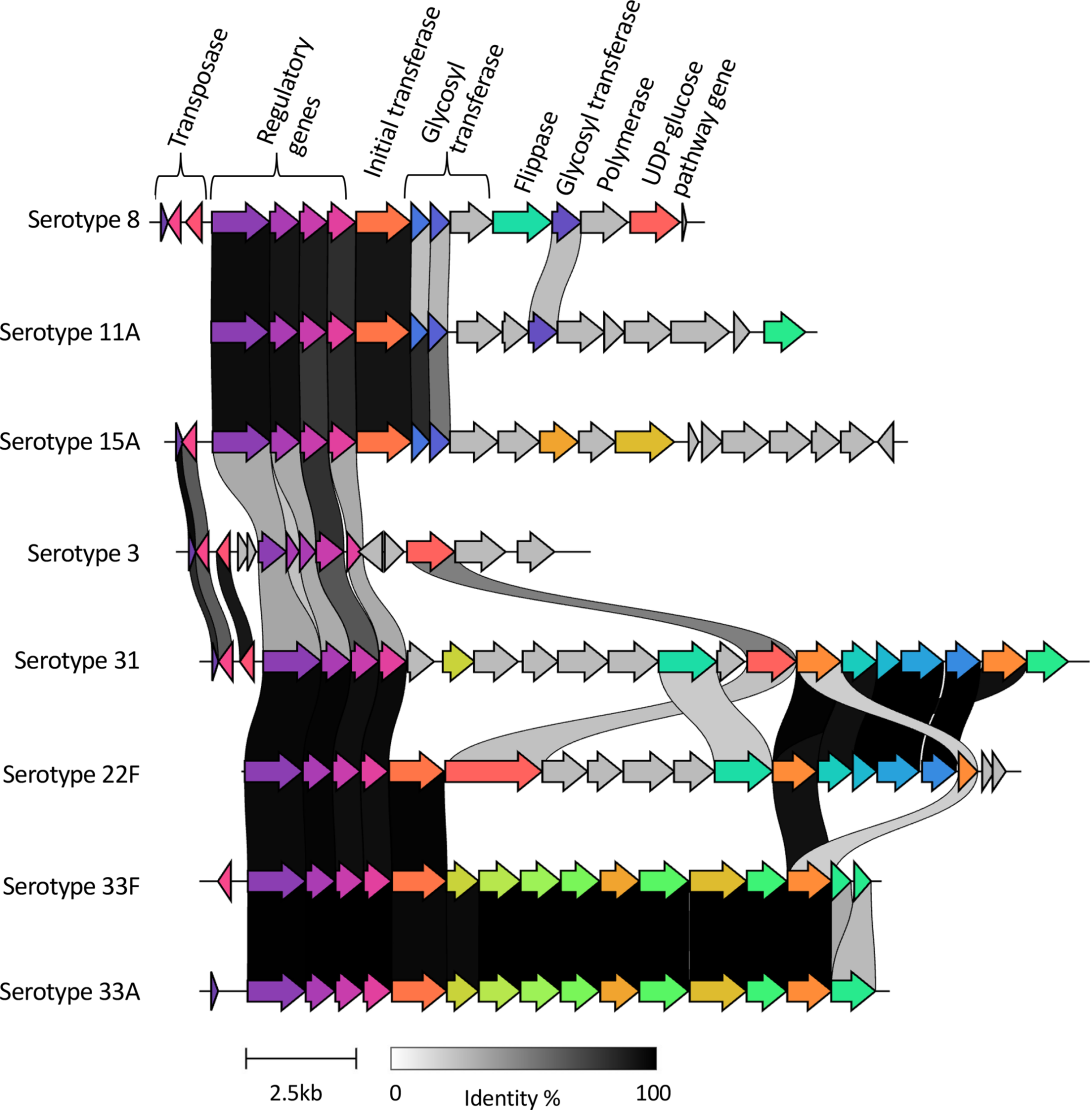
GPSC3 capsular loci comparative analysis. Eight representative capsular loci regions of each serotype belonging to GPSC3 are shown. The most recent (late-PCV13 period) representative genomes from the South African collection were selected for CPS extraction and analysis. Serotype-specific annotations of individual genes are shown in Fig. S5.

**Fig. 6.**
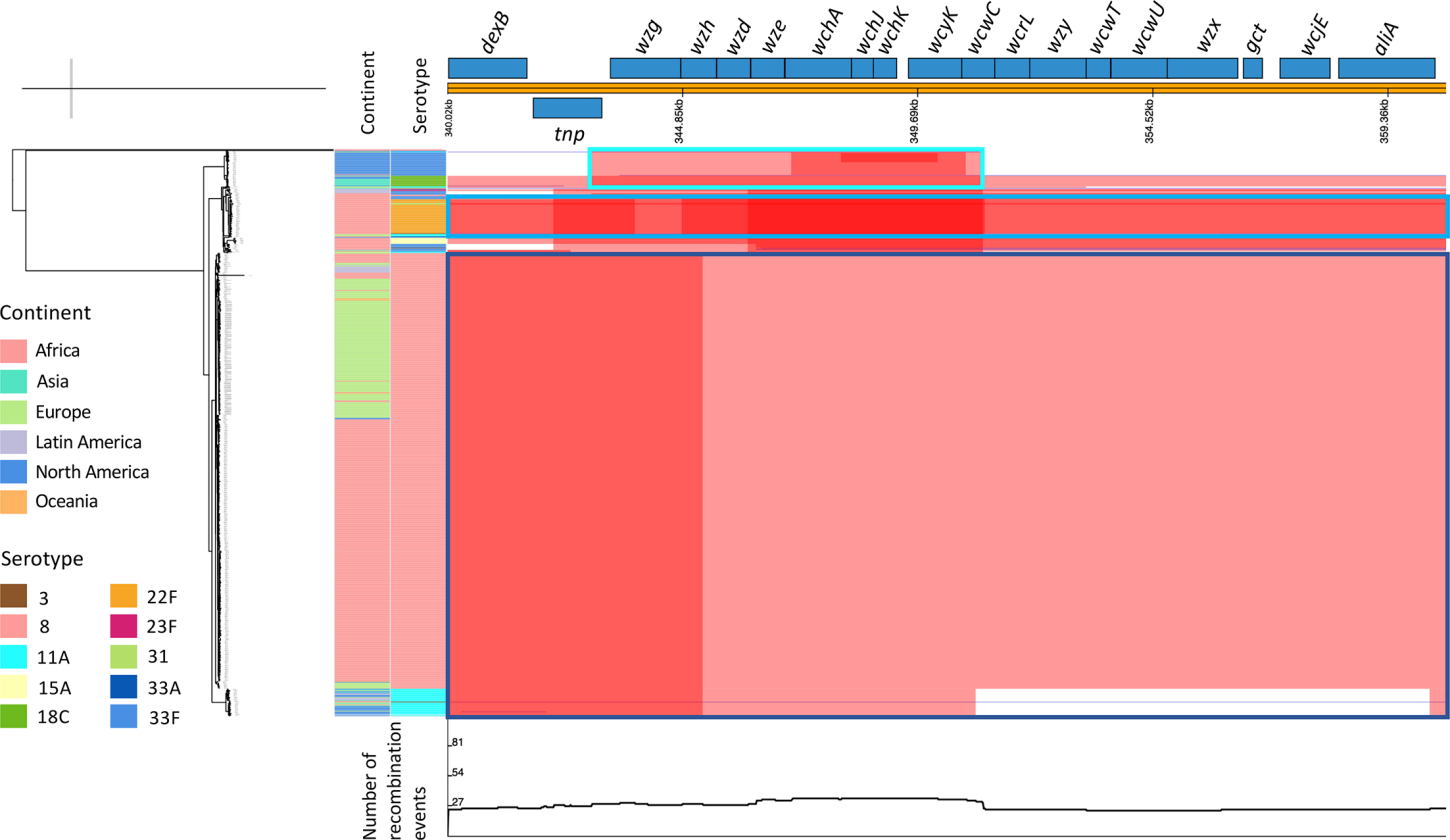
GPSC3 Phandango plot of recombination detected using Gubbins, focused on the *cps* locus including all GPSC3 isolates, *N*=749. The phylogenetic tree is on the left. Annotated GPSC3 reference (serotype 11A) is across the top. The recombination plot in the middle has red blocks showing recombination shared by multiple isolates in a clade; blue blocks showing recombination unique to a single isolate. Recombination blocks correspond with the genes across the reference. Overlapping recombination blocks increase the density of the colour. The bottom graph shows the number of recombination events affecting any single position in the reference. Serotype 8 and 11A recombination blocks are outlined in dark blue. The lighter blue outline highlights recombination among serotypes 22F and 31 isolates. The turquoise outline highlights recombination between serotype 18C and 33F isolates.

A global collection of 749 GPSC3 isolates expressed 10 serotypes, including non-PCV13 serotypes 8, 11A, 15A, 22F, 31, 33A and 33F (*n*=730), and PCV13 serotypes 3, 18C and 23F (*n*=19) (Fig. 4). Serotype 8 accounted for 77.6% (*n*=581) of the GPSC3 isolates. While GPSC3 largely expressed serotype 8 in Africa and Europe at frequencies of 83.6% (376/450) and 93.1% (188/202), respectively, GPSC3 predominantly expressed serotype 33F (57.1%, 32/56) and 11A (30.4%, 17/56) in North America (Fig. 4). There was no serotype 8 observation among the 16 GPSC3 isolates from Asia; however, serotype 11A (*n*=5) and 18C (*n*=9) accounted for 87.5% of the isolates, combined. Of the ten serotypes expressed by GPSC3 in this collection, GPSC3 isolates from South Africa (*N*=447) expressed all, except two (18C and 23F) of the isolates. Comparative analysis of the *cps* gene clusters of serotypes expressed by GPSC3 isolates from South Africa showed that the serotype 8 *cps* locus was closely related to that of 11A (GPSC3 reference) and 15A (Fig. 5). Annotated *cps* loci for each serotype are shown in Fig. S5. Recombination analysis of GPSC3 serotypes showed a major recombination block among serotype 8 isolates spanning the entire *cps* region, irrespective of isolate origin (Fig. 6). Similar recombination events were observed between serotype 8 and 11A isolates. We also observed other recombination events shared between serotypes. Serotype 22F and 31 from South Africa shared a recombination event that also spanned the *cps* region, with higher recombination densities from the *wzh* to *wcwC* genes (Fig. 6). Overall, serotype 8 had minimal recombination with other serotypes (Fig. 6).

*In silico* AMR predictions for GPSC3 (*N*=749) showed 100% susceptibility to penicillin (Fig. 4), similar to our serotype 8 phenotypic results. However, acquired resistance to erythromycin [3.9% (29/749)] conferred by *mefA* [86.2% (25/29)] and *ermB* [13.8% (4/29)], tetracycline resistance [2.1% (16/749)] conferred by *tetM* and cotrimoxazole resistance [7.9% (59/749)] by the presence of mutation I100L in *folA* and/or indel within amino acid residue 56–67 in *folP* were observed, more notably among the non-serotype 8 isolates expressed by GPSC3 (Fig. 4).

## Discussion

Our study shows that the increase in serotype 8 invasive disease in South Africa after the introduction of PCV was driven by the expansion of the pre-existing, globally disseminated GPSC3 lineage. Although serotype 8 (GPSC3, CC53) remains largely susceptible to commonly used antibiotics, it continues to cause invasive disease in both children and adults in South Africa after PCV introduction, with future predictions showing increases in the effective population size of the CC53 clone in the absence of serotype 8 targeting interventions. Serotype 8 isolates from South Africa showed clonality when contextualized in relation to isolates from other countries, suggesting that expansion was largely due to within-country transmission rather than transmission between countries. The GPSC3 lineage is diverse and expressed both vaccine and non-vaccine serotypes with varying geographical distribution and antimicrobial resistance profiles. This lineage also showed recombinogenic potential, with similar recombination signatures observed among serotypes 8 and 11A isolates.

The increase in non-vaccine serotype disease in both children and adults post-PCV vaccination in several countries continues to reduce the net effectiveness of PCVs [5,7,15]. The emergence and expansion of serotype 8 (GPSC3, CC53) after PCV introduction in South Africa and other settings demonstrate its continued successful propagation and niche occupation in the PCV era [9,14]. In South Africa, serotype 8 became the most common cause of invasive disease in 2015 (4 years after the introduction of PCV13) and accounted for 11% of overall IPD in our surveillance programme. By 2020, this proportion had reached 17% (data not shown). Several studies have shown that the post-PCV increase in non-vaccine serotype IPD was due to a limited number of non-vaccine serotypes, with serotype 8 being a major contributor. Data from long-term surveillance in France and Spain have shown substantial increases in serotype 8 IPD in children and adults after PCV introduction [42,43]. The European Centre for Disease Prevention and Control reported a 120% increase in the frequency of serotype 8 IPD in EU/EEA countries in 2017 [44]. These findings were further corroborated by an international study on trends in invasive bacterial diseases showing serotype 8 consistently being the leading cause of invasive disease among *S. pneumoniae* cases from 2018 to 2021 [45].

Serotype 8 is relatively less frequently detected in paediatric carriage and is occasionally associated with outbreaks [46,48]. A meta-analysis of data collected from 20 locations across Africa, Europe, Latin America and North America found that among 25 pneumococcal serotypes, serotype 8 had the fifth highest invasive disease potential estimate after PCV13 serotypes 1, 5, 7F and non-vaccine serotype 12F [49]. In France, serotype 8 together with emerging non-PCV13 serotypes 12F, 24F and 33F (also known to have high invasive disease potential), frequently caused bacteraemic pneumonia in patients without underlying conditions [49,50], supporting invasive potential advantages. We observed different clinical presentations for serotype 8 IPD among children and adults. A rat model study showed evidence of serotype 8 (ST53) hypervirulence, causing brain injury with a high mortality rate compared to vaccine-serotype 14 and non-vaccine serotype 15B [51]. This may influence resistance to opsonophagocytosis among children due to immature immune systems, resulting in increased likelihood of central nervous system invasion, causing meningitis as opposed to bloodstream invasion and mucosal persistence in adults.

The CC53 clone of serotype 8 is among the most successful clones in the post-PCV era globally. Our CC53 phylodynamic analysis showed that the CC53 clone had at least five clonal expansions in the past decades, with the most recent clonal expansion occurring between 1990 and 1994, before PCV use. Predicted future population dynamics showed continued increase in the effective population sizes of CC53 subpopulations over the next 10 years, at least, starting from 2020, suggesting potential for continued expansion. Amid global expansion and dissemination, CC53 isolates from South Africa exhibited distinct clustering, implying expansion was largely driven by within-country circulation, although two isolates from neighbouring country Mozambique clustered within the South African clade. Clustering of the South African CC53 clade was independent of demographic or clinical characteristics. The serotype 8 *cps* locus was largely conserved within the global collection. However, we observed variations within certain *cps* locus genes such as the *wchA* gene, which encodes the initial transferase essential for capsular polysaccharide synthesis [52]. While certain variations in *wchA* in serotype 8 isolates were associated with loss in capsule expression [53], in the current study, all serotype 8 isolates that allowed for *wchA* sequence variation were typeable by Quellung reaction. These variations concur with serotype 8 (CC53) findings from Spain, where mutations, particularly in the *wchA* gene in some variants, led to increased adhesion to the lung epithelium and biofilm formation [54], features that could explain the predominance of this clone.

Although serotype 8 (GPSC3) remains largely susceptible to antimicrobials, it continues to be a major cause of IPD, especially adult IPD in many European countries [5,14, 46], and a leading cause of invasive disease among South African children and adults [9,11]. Additionally, the possibility of developing antimicrobial resistance exists. For example, a multidrug-resistant recombinant clone of serotype 8 (ST63) that arose due to recombination between a donor Netherlands 8-ST53 clone and a recipient Sweden 15A-ST63 clone expanded in Spain after the initial use of PCV in 2001, with prominent spread in 2005 [55]. Similarly, we observed macrolide-nonsusceptibility among GPSC3-expressed non-vaccine serotype 11A (CC62) and 33F (CC100, ST717 and ST2705) at serotype-specific proportions of 19% and 37%, respectively. Our results showed that the serotype 8 *cps* locus was closely related to both serotype 11A and 15A. GPSC3 has previously been shown to have a lower than average recombination rate (r/m of 5.33 vs. median r/m of 7.78, 95% CI 6.81–8.71) as determined from 60 dominant GPSCs using the Wilcoxon rank sum test [24]. However, our study showed evidence of CPS region recombination signatures within this lineage with similar recombination events observed among serotypes 8 and 11A isolates. The exploration of genome-wide recombination events may be useful for this lineage to uncover insights into potential resistance acquisition. Nonetheless, this work, including several other studies, shows that serotype 8 is an established strain that has successfully persisted and continues to cause IPD despite remaining largely susceptible to commonly used antimicrobials.

This study had some limitations. We randomly sampled ~300 isolates per year without adjusting for the significant decline in vaccine-serotypes following PCV use and, therefore, might have oversampled non-vaccine serotypes, including serotype 8 in the post-PCV13 era. Serotype 8 and GPSC3 incidences were determined using the total number of cases with serotyping data from our GERMS-SA surveillance programme and did not account for those without serotyping data, although missing serotyping data was assumed to have occurred at random. We also note that serotype 8 incidence is an underestimation, as we did not correct for IPD under-reporting. We used a subset of serotype 8 genomes from South Africa to describe circulating lineages and, therefore, may have missed emerging or rare serotype 8 lineages. Additionally, as in our surveillance programme, province-level representation was skewed, and, therefore, serotype 8 distribution and its genomic variants were limited to the country level. In the global collection, serotype 8 and GPSC3 isolates were not systematically selected in some settings, and we did not include GPSC3 genomes outside the GPS project, which limited our ability to infer lineage predominance in other settings compared to South Africa. Among isolates from Africa, there was considerable underrepresentation of serotype 8 genomes. Nonetheless, our genomic findings were comparable to those from Denmark and Spain [13,14], identifying serotype 8 GPSC3 (CC53) as the most prevalent disease-causing lineage post-PCV, with other serotype 8 lineages having little disease contribution.

Serotype 8 has substantially contributed to non-vaccine serotype invasive disease in both children and adults in the post-PCV era, globally, while remaining largely susceptible to first-line treatment. The reasons behind this successful propagation are not fully understood. Serotype 8 is targeted by the PCV20 formulation that is recommended for use in the USA and other European countries, and because the CPS locus of serotype 8 is largely conserved, chances of vaccine escape may be low. South Africa switched to a lower-valency, but affordable PCV10 (Pneumosil, Serum Institute of India) formulation in 2024, which does not include serotype 8. Although transitioning to the lower valency PCV10 may be unlikely to directly affect serotype 8 disease trends, it may facilitate the continued expansion of this serotype following potential changes in indirect vaccine effects and reduced competitive pressure from other circulating serotypes. Therefore, continued surveillance will be particularly important to monitor the impact of this switch on serotype, lineage and AMR distributions and may inform the use of higher valency PCVs that target this serotype or the inclusion of serotype 8 in lower valency formulations.

## Supplementary material

10.1099/mgen.0.001737 Supplementary Material 1.

10.1099/mgen.0.001737 Supplementary Material 2.

## Abbreviations

AST: antimicrobial susceptibility testing
CC: clonal complex
CI: confidence intervals
CI: Credible interval
CPS: capsular locus sequences
CSF: cerebrospinal fluid
GPS: global pneumococcal sequencing
GPSC3: global pneumococcal sequence cluster 3
GPSC: global pneumococcal sequence cluster
GTR: generalized time-reversible
IPD: invasive pneumococcal disease
IRR: incidence rate ratio
MCMC: Markov chain Monte Carlo
MICs: minimum inhibitory concentrations
MLST: multi-locus sequence type
NICD: National Institute for Communicable Diseases
non-PCV13: non-13-valent pneumococcal conjugate vaccine
PCV: pneumococcal conjugate vaccine
PCV7: seven-valent pneumococcal conjugate vaccine
