# Supplementary material for: Clonal expansion of global pneumococcal sequence cluster 3 within serotype 8 after 13-valent pneumococcal conjugate vaccine introduction, South Africa
Source: Microb Genom. 2026 Jun 2;12(6):001737. doi: 10.1099/mgen.0.001737 (PMC13229004; doi:10.1099/mgen.0.001737)
Supplement: Supplementary Material 1. [file mgen-12-01737-s001.pdf]

Supplementary tables and figures

Table S1. Incidence rate ratio of invasive disease caused by serotype 8 pneumococci by vaccine periods stratified by age group.

	**Incidence per 100,000 population**		**Incidence rate ratio**	
	Pre-PCV (2005-2008)	Late-PCV13 (2015-2020)	IRR (95% CI)	Two-sided p-value
0-4y	0.4	0.9	2.3 (1.3 - 4.1)	<0.01
5-14y	0.06	0.06	1.0 (0.3 - 3.7)	1.0
15-24y	0.06	0.1	1.8 (0.6 - 5.9)	0.3
25-44y	0.4	0.4	1.1 (0.7 - 1.5)	0.8
45-64y	0.3	0.6	2.1 (1.2 - 3.7)	<0.01
>64y	0.2	0.7	2.8 (1.1 - 8.3)	0.02

Table S2. Serotype 8 isolates by country, vaccine period, source of specimen, and age group.

Country	Vaccine introduction year	n	Vaccine period		Source of specimen		Age category	
			Pre-PCV n (%)	Post-PCV n (%)	Disease n (%)	Carriage n (%)	<5 years n (%)	≥5 years n (%)
Bangladesh	2015 (PCV10)	1	1 (100)	0 (0)	1 (100)	0 (0)	1 (100)	0 (0)
Belarus	2014 (PCV10)	2	0 (0)	2 (100)	0 (0)	2 (100)	1 (50)	1 (50)
Brazil	2010 (PCV10)	10	2 (20)	8 (80)	10 (100)	0 (0)	4 (40)	6 (60)
Bulgaria*†	No PCV‡	1	_	_	1 (100)	0 (0)	_	_
China	2009 (PCV7); 2010 (PCV10); 2011 (PCV13)	1	0 (0)	1 (100)	0 (0)	1 (100)	1 (100)	0 (0)
Egypt*	No PCV‡	1	_	_	1 (100)	0 (0)	1 (100)	0 (0)
Ethiopia*†	No PCV‡	1	_	_	0 (0)	1 (100)	_	_
France†	2006 (PCV7); 2010 (PCV13)	1	1 (100)	0 (0)	1 (100)	0 (0)	_	_
India◇✧†	2017 (PCV13)	10	5 (50)	4 (40)	9 (90)	_	1 (10)	8 (80)
Israel	2009 (PCV7); 2010 (PCV13)	11	3 (27)	8 (73)	11 (100)	0 (0)	8 (73)	3 (27)
Malawi†	2011 (PCV13)	5	2 (40)	3 (60)	4 (80)	1 (20)	3 (60)	1 (20)
Malaysia*	No PCV‡	2	_	_	2 (100)	0 (0)	0 (0)	2 (100)
Mongolia*†	No PCV‡	1	_	_	1 (100)	0 (0)	_	_
Morocco	2010 (PCV13); 2012 (PCV10)	1	1 (100)	0 (0)	1 (100)	0 (0)	1 (100)	0 (0)
Mozambique†	2013 (PCV10)	3	3 (100)	0 (0)	3 (100)	0 (0)	2 (67)	1 (33)
Nepal	2015 (PCV10)	13	8 (62)	5 (39)	3 (23)	10 (77)	11 (85)	2 (15)
Netherlands✧	2011 (PCV10)	178	43 (24)	135 (76)	_	_	5 (3)	173 (97)
New Zealand	2008 (PCV7); 2011 (PCV10); 2014 (PCV13)	5	0 (0)	5 (100)	5 (100)	0 (0)	5 (100)	0 (0)
Nigeria	2014 (PCV10)	1	0 (0)	1 (100)	1 (100)	0 (0)	1 (100)	0 (0)
Papua New Guinea*	No PCV‡	6	_	_	6 (100)	0 (0)	6 (100)	0 (0)
Poland*	No PCV‡	2	_	_	2 (100)	0 (0)	2 (100)	0 (0)
Russian Federation	2014 (PCV13)	5	5 (100)	0 (0)	5 (100)	0 (0)	0 (0)	5 (100)
Slovenia*	No PCV‡	2	_	_	2 (100)	0 (0)	2 (100)	0 (0)
South Africa	2009 (PCV7); 2011 (PCV13)	391	30 (8)	361 (92)	391 (100)	0 (0)	218 (56)	173 (44)
Thailand ▲	No PCV‡	2	_	_	2 (100)	0 (0)	0 (0)	2 (100)
The Gambia*	2009 (PCV7); 2011 (PCV13)	10	0 (0)	10 (100)	1 (10)	9 (90)	3 (30)	7 (70)
Turkey	2008 (PCV7)	1	0 (0)	1 (100)	1 (100)	0 (0)	0 (0)	1 (100)
United States	2000 (PCV7); 2010 (PCV13)	19	2 (11)	17 (90)	19 (100)	0 (0)	7 (37)	12 (63)

‡ No vaccine introduction
*Vaccine period data not included
◇Some or all isolates missing year information
†Some or all isolates missing age information
✧ Some or all isolates missing source information
▲ PCV10 and PCV13 are available in Thailand, but not included in the National Immunization Programme

Table S3. Distribution of lineages expressing serotype 8 by continent and country of origin.

Continent	GPSC lineages									
Country	3 (n=581)	22 (n=1)	32 (n=4)	98 (n=47)	224 (n=34)	277 (n=10)	336 (n=6)	694 (n=2)	732 (n=1)	Total (n=686)
Africa	376	1	-	22	9	5	-	-	-	413
Egypt	-	-	-	1	-	-	-	-	-	1
Ethiopia	-	-	-	-	1	-	-	-	-	1
Malawi	-	-	-	1	-	4	-	-	-	5
Morocco	1	-	-	-	-	-	-	-	-	1
Mozambique	2	-	-	1	-	-	-	-	-	3
Nigeria	-	-	-	1	-	-	-	-	-	1
South Africa	373	1	-	10	6	1	-	-	-	391
The Gambia	-	-	-	8	2	-	-	-	-	10
Asia	-	-	-	-	17	5	6	1	1	30
Bangladesh	-	-	-	-	1	-	-	-	-	1
China	-	-	-	-	1	-	-	-	-	1
India	-	-	-	-	-	4	5	-	1	10
Malaysia	-	-	-	-	1	-	-	1	-	2
Mongolia	-	-	-	-	1	-	-	-	-	1
Nepal	-	-	-	-	11	1	1	-	-	13
Thailand	-	-	-	-	2	-	-	-	-	2
Europe	188	-	4	10	-	-	-	1	-	203
Belarus	1	-	-	1	-	-	-	-	-	2
Bulgaria	1	-	-	-	-	-	-	-	-	1
France	1	-	-	-	-	-	-	-	-	1
Israel	9	-	-	2	-	-	-	-	-	11
Netherlands	169	-	1	7	-	-	-	1	-	178
Poland	2	-	-	-	-	-	-	-	-	2
Russian Federation	2	-	3	-	-	-	-	-	-	5
Slovenia	2	-	-	-	-	-	-	-	-	2
Turkey	1	-	-	-	-	-	-	-	-	1
Latin America	9	-	-	1	-	-	-	-	-	10
Brazil	9	-	-	1	-	-	-	-	-	10
North America	3	-	-	14	2	-	-	-	-	19
United States	3	-	-	14	2	-	-	-	-	19
Oceania	5	-	-	-	6	-	-	-	-	11
New Zealand	5	-	-	-	-	-	-	-	-	5
Papua New Guinea	-	-	-	-	6	-	-	-	-	6

Table S4. Counts of MLST clonal complexes (CC) and sequence types (ST) by Global Pneumococcal Sequence Cluster (GPSC).

GPSC<br>CC/ST	Count
3	581
CC1012	2
CC53	579
22	1
ST17734	1
32	4
CC2331	1
ST2331	2
ST944	1
98	47
CC1480	29
CC3406	15
ST2894	2
ST4202	1
224	34
ST16229	1
ST3500	3
ST3714	1
ST4216	16
ST6022	8
ST6748	5
277	10
CC10588	9
ST15546	1
336	6
CC12793	5
ST14431	1
694	2
ST5039	2
732	1
ST8254	1
Total	686

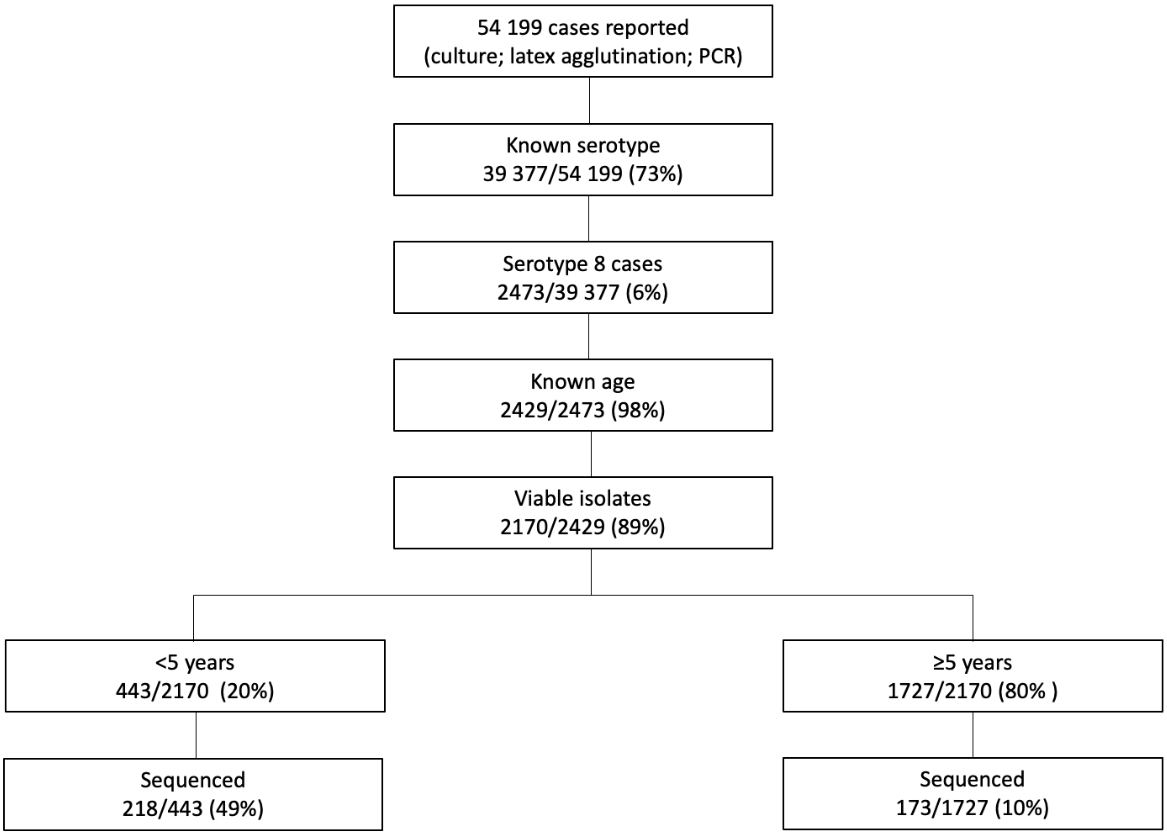

Figure S1. Summary of serotype 8 isolates that were whole-genome sequenced among the <5 and ≥5 years, South Africa, 2005–2020, N=391.

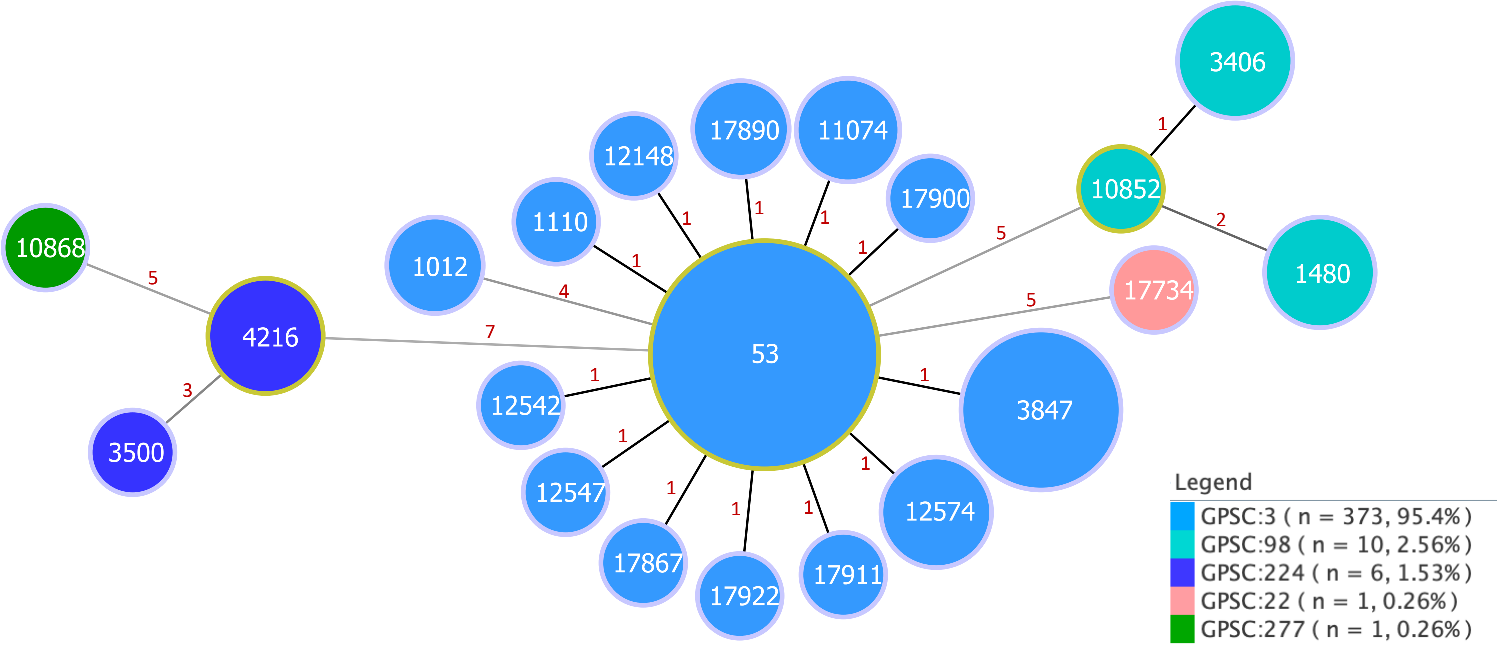

Figure S2: Multilocus sequence typing (MLST) minimum-spanning-tree of all sequenced South African isolates (n=391, 2005–2020). The size of each circle is proportional to the number of isolates within each sequence type (ST). ST relatedness is shown by the joining lines with allelic differences represented by the red numbers. The isolates were grouped using the single-locus variant MLST scheme. The circles are coloured by Global Pneumococcal Sequence Clusters (GPSCs).

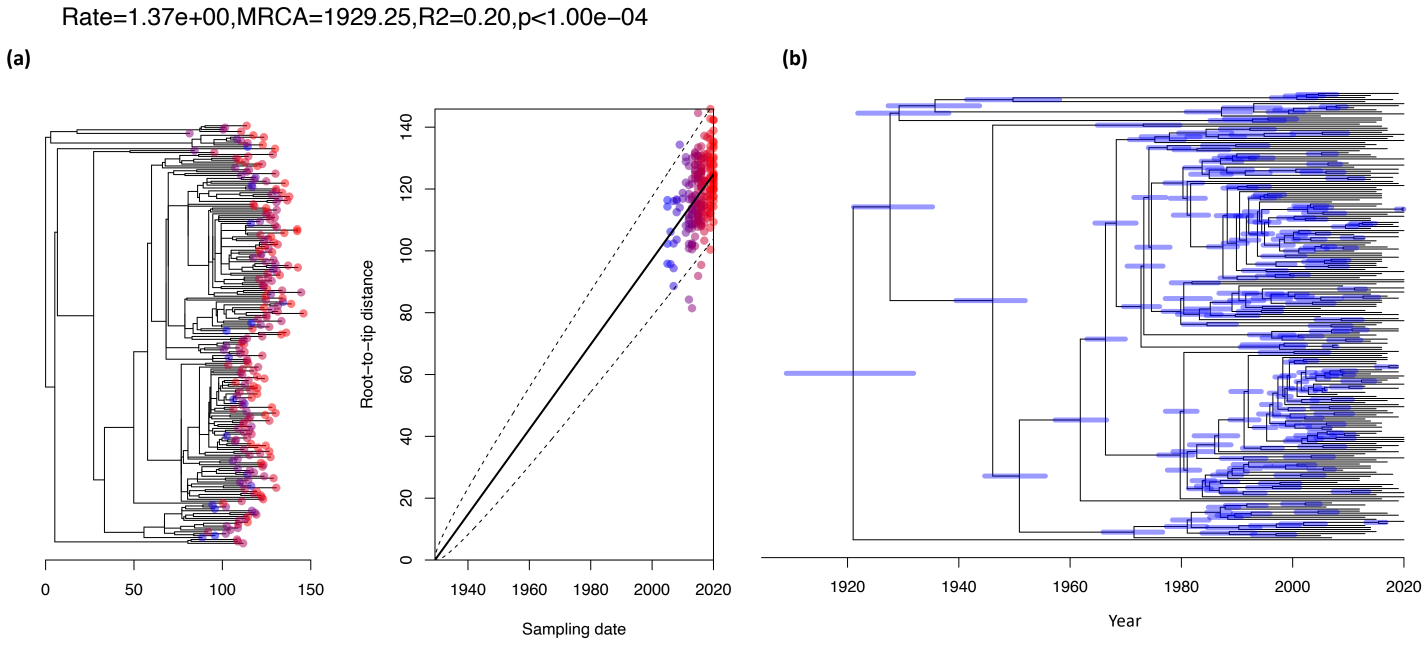

Figure S3: Clonal complex 53 (CC53) Bayesian inference of ancestral dates using BactDating among South African isolates (N=219), 2005–2020. a) Root-to-tip distance regression analysis for temporal signal detection of recombination-free CC53 phylogeny. Tips are shaded in a colour gradient from red (more recent isolates) to blue (older isolates). b) Time-resolved phylogeny showing ancestral dates with 95% credible intervals represented by blue shades.

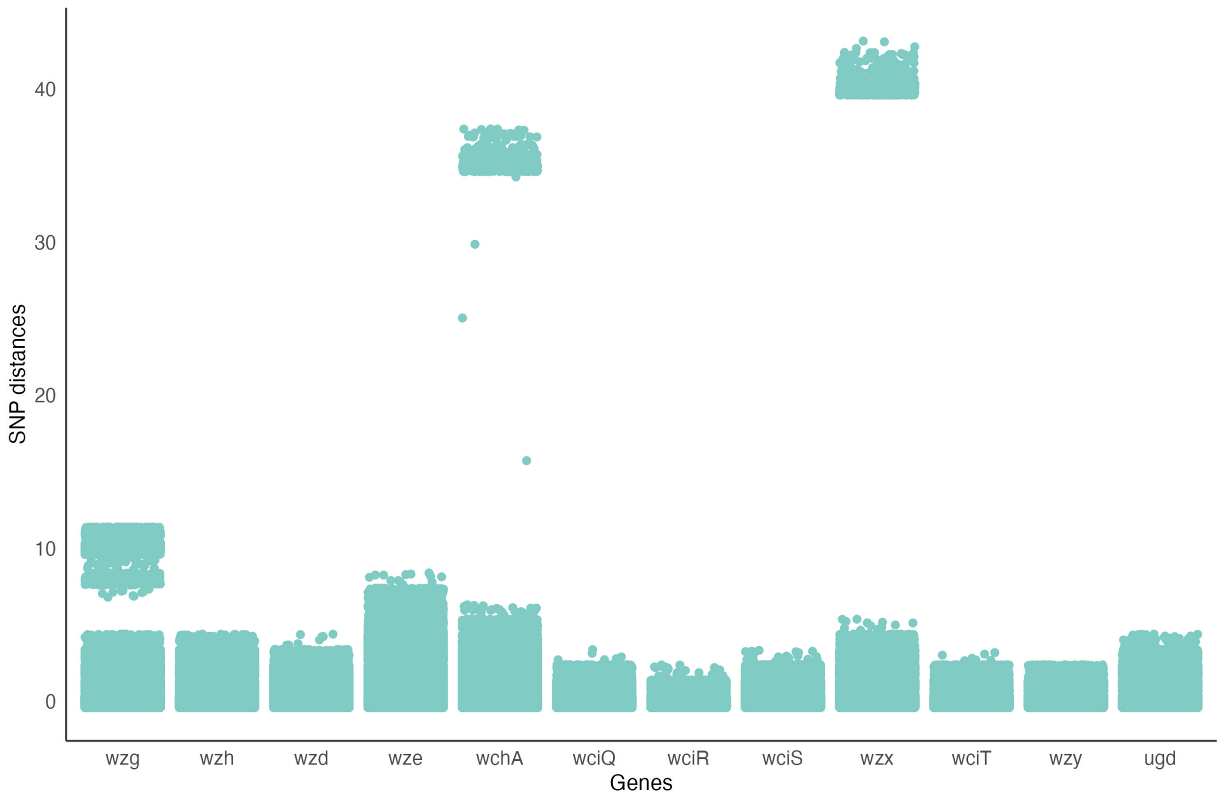

Figure S4: Serotype 8 capsular locus (CPS) single nucleotide polymorphism (SNP) analysis, N=686. SNP variations by gene from the serotype 8 reference sequence (CR931644) are expressed by SNP distances.

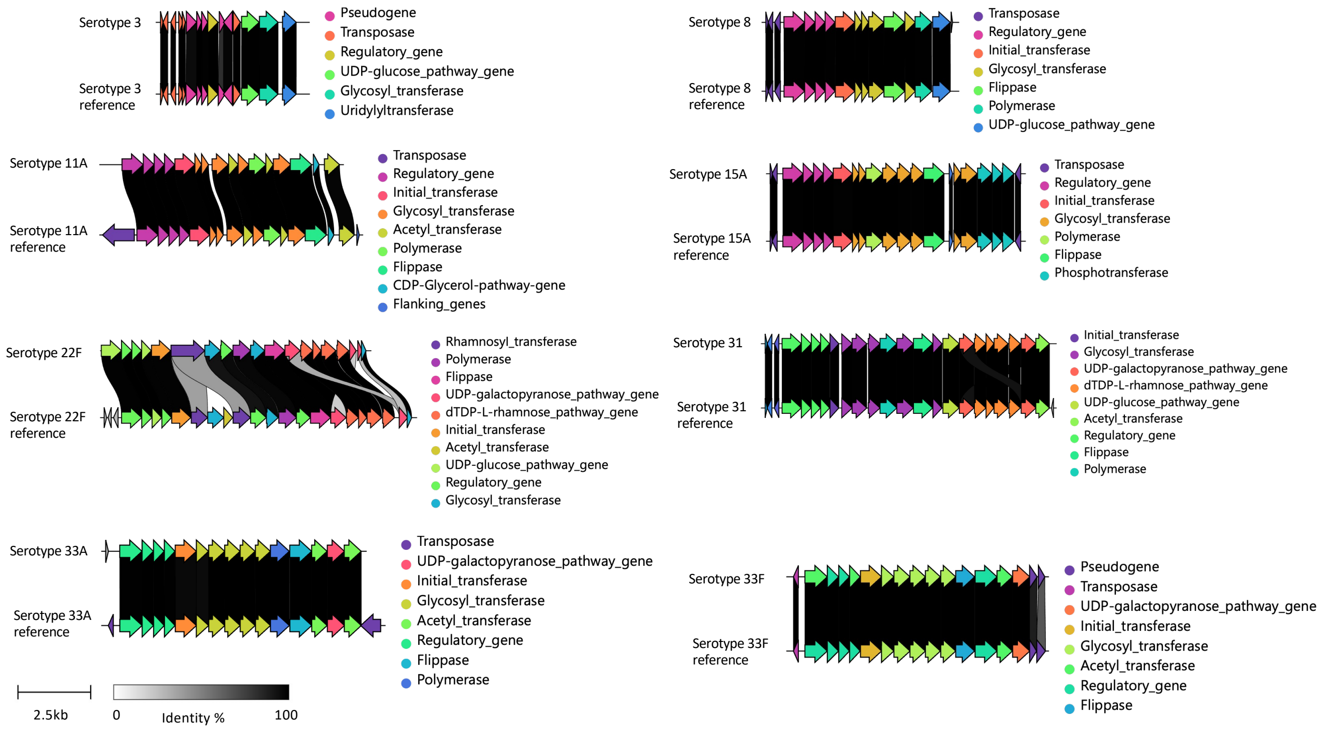

Figure S5: Annotated gene clusters of capsular loci (CPS) of serotypes expressed by GPSC3. Each serotype is aligned against its reference.
